# Inhibition Ability of Food Cues between Successful and Unsuccessful Restrained Eaters: A Two-Choice Oddball Task

**DOI:** 10.1371/journal.pone.0120522

**Published:** 2015-04-17

**Authors:** Fanchang Kong, Yan Zhang, Hong Chen

**Affiliations:** 1 Key Laboratory of Adolescent CyberPsychology and Behavior (Ministry of Education) and School of Psychology, Central China Normal University, Wuhan, China; 2 Key Laboratory of Cognition and Personality (Ministry of Education) and Faculty of Psychology, Southwest University, Chongqing, China; 3 Academy of Educational Science, Huazhong University of Science & Technology, Wuhan, China; University of Florida, UNITED STATES

## Abstract

**Background:**

Previous studies have presented mixed findings on the inhibition ability in restrained eaters (REs) due to the limited amount of neural evidence and limitations of behavioral measures. The current study explores the neural correlations of the specific inhibition ability among successful restrained eaters (S-REs), unsuccessful restrained eaters (US-REs), and unrestrained eaters (UREs).

**Methodology and Principal Findings:**

Three groups of females (with 13 participants in each group) completed a two-choice Oddball task, while the event-related potentials (ERPs) are recorded synchronously. Results indicate that S-REs showed inhibition deficit in processing high-energy food cues whereas US-REs show inhibition deficit in processing both low- and high-energy food cues.

**Conclusion:**

Results indicate that S-REs and US-REs differ in terms of specific inhibition ability and that enhanced inhibition is essential to a successful diet.

## Introduction

Self-control is defined as the exertion of control over the self by the self [1], which reflects the ability to change one’s responses, and accommodates an individual’s reaction to the behavioral standard to support the pursuit of a long-term goal [2]. Numerous studies have shown that self-control and self-inhibition have significant roles in successful weight control [3, 4] and food intake [5]. Longitudinal studies confirmed that initial inhibition ability predicted weight gain after one year [6] and overweight after three years [7]. Food exposure successfully evoked self-regulation in normal-weight REs but not for the overweight REs because the latter ignored the diet goal [8]. Therefore, systematically examining the inhibition ability of REs would be beneficial in uncovering the mechanism of a successful diet.

Behavioral studies showed that compared with UREs, REs reported a higher level of compulsive or lower level of inhibition ability [9]. Participants in the impulsive group had a significantly higher caloric intake during a subsequent taste test, whereas those under the inhibition group did not differ from the control group. Therefore, impulsivity is a direct cause of overeating [10]. Women who scored higher in uncontrolled eating (TFEQ-D) were significantly more impulsive on the Barrett Impulsivity Scale-11 (BIS-11), but not on the delay discounting task (DDT) and the go/no-go task. Meanwhile, the women who exhibited higher restraint in eating (TFEQ-R) scores were significantly less impulsive on the go/no-go, task but did not differ in terms of the BIS-11 and the DDT. Therefore, some aspects of overeating may be caused by a poor ability to reflect on decisions [11]. High REs did not inhibit their eating behaviors when faced with highly palatable food and appeared to overeat, a reaction that is expected to result in obesity [12].

Newer studies showed that the interaction between dietary restraint and trait impulsiveness predicted dieting success. Specifically, a lower level of impulsiveness is associated with greater dieting success among REs, suggesting that less impulsive REs are more likely to become S-REs [13]. However, some studies have reported contrasting findings. For example, REs commit fewer errors compared with UREs regardless of food or nonfood cues [5]. Meule, Vüele, and Kübler (2012) showed that REs did not differ from UREs in terms of resistance to distractor interference, although REs reacted faster to the high-calorie food cues than the neutral pictures. This result indicates that REs demonstrate an attention bias toward high-calorie food cues, which is related to low dieting success [14]. Furthermore, the latest studies showed that impulsive reactions to high-calorie food cues are pronounced when both trait impulsivity and food craving are high. However, low levels of impulsivity can compensate for high levels of trait food craving [15]. Thus, REs probably showed inhibition deficit in the specific stimuli (food cues), but not in the general stimuli.

Some studies used cognitive neuroscience technology, such as event-related potentials (ERPs), to examine the neural correlates of the REs’ inhibition ability. Hachl, Hempel, and Pietrowsky (2003) found that REs showed no significant difference in neural correlates, but not in behavioral response. Specifically, larger P2 amplitude of REs was comparable to UREs in the hungry condition, but larger P2 amplitude was comparable to UREs in full condition. In addition, larger late positive potential (LPP) for REs was comparable to UREs in both food and nonfood conditions [16], suggesting that REs require more cognitive resources to inhibit the interference of unrelated information in processing a target stimuli. In another ERP study, the REs and contrast groups completed two blocks [17]. In the first block, emotional pictures and pictures of high-energy food were presented, and the participants were informed that they were allowed to eat half of the food presented in the first block and that the other half was not available by manipulating the availability. Results showed no significant differences between the REs and the contrast group in the first block. However, a significant difference was observed in the second block. For REs, the non-available food cues elicited more positive LPP than the available food cues, but not for the control group. Therefore, the REs successfully conducted cognitive control on the motivation tendency based on the motivational background to reduce the sensibility to the food cues and attain affirmative results in dieting.

Furthermore, Babiloni, Del Percio, Triggiani, Marzano, Valenzano, Petito et al. (2011) used the standard oddball task to explore the temporal processing between the normal-weight successful dieters and non-dieters. The results showed that larger frontal-parietal P3 were evoked by food pictures among normal-weight successful dieters than in non-dieters [18]. A new study, which employed a go/no-go task to investigate the neurocognitive correlates of processing food-related stimuli, showed that food images elicited significantly enhanced N2, P3, and slow-wave ERP components compared with the nonfood images, suggesting that processing food-related stimuli elicited distinct patterns of cortical activity [19]. Therefore, a probable difference existing between REs and UREs in terms of the neural correlates of processing food stimuli, such as P2, N2, and P3 components.

In summary, the existing literature showed contrasting results in inhibition ability of REs to food cues importantly because limited neural studies were found, specifically on the dynamic temporal process in REs. Previous studies showed that REs comprise successful restrained eaters (S-REs) and unsuccessful restrained eaters (US-REs) [4, 5, 20]. The difference between the neural mechanism of S-REs and US-REs showed significant risks for obesity and other eating-related problems [21]. Therefore, the present study investigated the neural correlates of inhibition ability to food cues in a two-choice oddball task using high time-resolution ERPs. Considering the abovementioned findings, we hypothesized that S-REs and US-REs differed in N2, P2, and P3 components compared with UREs. Furthermore, S-REs and US-REs showed stronger N2, P2, and P3 components in processing food cues compared with neural cues.

## Method

### Ethics Statement

This study has been approved by the Ethics Committee of the Faculty of Psychology of Southwest University. All participants have signed an informed consent prior to their inclusion in our experiments. The study was designed in accordance with tenets of the Declaration of Helsinki.

### Participants

The final sample included 13 S-REs, 13 US-REs, and 13 UREs from Southwest University in Chongqing. Based on the operationalization described in previous studies [22], the S-REs who scored more than 2.7 on the restrained eating subscale of the Dutch Eating Behavior Questionnaire (DEBQ), less than 3.0 on the emotional and external eating subscales of the DEBQ, less than 1.8 on the uncontrolled eating subscale of the Three-Factor Eating Questionnaire (TFEQ), and had normal BMI for more than six months [23].US-REs scored more than 2.7 on the Restrained Eating subscale of DEBQ, more than 3.0 on the Emotional Eating subscale and External Eating subscale of DEBQ, more than 1.8 in the Uncontrolled eating subscale of TFEQ, with had a normal BMI for more than 6 months. UREs never or infrequently dieted, scored less than 1.6 on the Restrained Eating subscale of DEBQ, with normal BMI (Table 1).

**Table 1:** Descriptive statistics and ANOVA analysis on the grouping variables.

Variable	Group	M ± SD	F & *p* value
BMI	S-RE	20.22±1.88	
	US-RE	19.62±2.05	F_(2,39)_ = 0.335, *p* =. 718
	URE	19.89±1.92	
DEBQ_R	S-RE	26.29±3.05	
	US-RE	26.57±10.48	F_(2,39)_ = 21.485, *p* =. 001
	URE	10.50±6.80	
DEBQ_EM	S-RE	20.64±±8.62	
	US-RE	33.57±16.89	F_(2,39)_ = 3.559, *p* =. 038
	URE	22.07±15.28	
TFEQ_U	S-RE	16.93±3.03	
	US-RE	20.86±2.38	F_(2,39)_ = 8.937, *p* =. 007
	URE	15.71±4.38	

Note: BMI = body mass index, DEBQ_R = Restrained Eating subscale of Dutch Eating Behavior Questionnaire, DEBQ_EM = Emotional and External Eating subscales of DEBQ, TFEQ_U = Uncontrolled eating subscale of Three Factor Eating Questionnaire.

All participants were healthy right-handed young women ranging in age from 18 to 23 (M = 19.3, SD = 0.3) and they were Han nationality. None of them reported having physical or psychiatric conditions, an eating disorder, or taking medication, smoking recreational drug or alcohol consumption during the past two years. Furthermore, all participants had a body mass index (BMI) within the average range (M = 20.13, SD = 1.05), and no significant difference between S-REs, US-RE and UREs (F_(2,39)_ = 0.335, *p* =. 718). Participants were paid 50 RMB for their participation.

### Materials and procedure

A two-choice Oddball task was employed in the second experiment [24]. Standard stimulus was a picture of steel clock, deviant stimuli included 30 high-energy food pictures, 30 low-energy food pictures and 30 neutral pictures [22]. High-energy food pictures differed from low-energy food pictures in the dimension of food content (*t*_(57)_ = 23.472, *p* =. 001), but not in the dimensions of arousal (F_(2,87)_ = 0.239, *p* =. 788), happiness (F_(2,87)_ = 2.162, *p* =. 121) and familiarity (F_(2,87)_ = 2.767, *p* =. 068). Each picture was identical in size (400 by 400 pixels), resolution (72 dots per inch), brightness, and background. Additional 15 pictures (five for each category) were selected in the practice session and not used in the normal session.

There were 6 blocks, with 120 trials in each block. In each block, stand stimuli was 75 percent and deviant stimuli was 25 percent. Trials in each block were presented randomly. In each trial, a black fixation appeared 300 ms in the gray screen, followed 500–1000 ms grey screen, subsequent target picture presented 1200 ms, and finally a grey screen appeared 1000ms before the next trial. In each group, half of the participants were required to press the “F” key when the standard stimuli presented, and press the “J” key when the deviant stimuli presented. Half of participants were asked to press the opposite key when the standard or deviant stimuli presented [25]. The distance from subjects to screen center was 0.9 m, the visual angle of the participant was less than 6°. Levels of hungry/fullness and emotional state were measured by self-report in the Likert scale from “0” (not at all) to “100” (very much) pro- and post-experiment. Moreover, the participant was not required to eat any food but water (250ml) within 3 hours prior to the study.

### ERP recording and analysis

EEG was recorded from 64 scalp sites using tin electrodes mounted in an elastic Ag/AgCl cap, with the linked reference on the left and right mastoids, and a ground electrode was placed on the medial frontal aspect (Brain products, German). Eye movements were monitored with supra- and infra-orbital electrodes and with electrodes on canthi. A bandpass of 0.01-100Hz was used for EEG and electro-oculogram (EOG). Electrode impedance was maintained below 5 kΩ. After rejecting trials with eye movements, blink, motion or other artifacts at each channel, the averaging of ERPs was computed off-line with computer algorithms. Trials with EOG artifacts with peak-to peak deflection exceeding ±80μV and those contaminated with artifacts were excluded from averaging.

In the experiment, only responses to the deviant stimuli were analyzed due to our hypothesis. The resulting grand averages were based on the correct trials and the averaging epoch was 1000 ms including a 200 ms pre-onset baseline. According to available findings[24,26] and the grand averaging ERPs, high-energy food pictures, low-energy food pictures and neutral pictures were significantly different in the P2 (130–205 ms), N2 (210–285 ms) and P3 (300–500 ms) components. The following cortexes were selected, frontal (F3, F4, Fz), frontal-central (FC3, FC4, FCz), central (C3, C4, Cz), central-parietal (CP3, CP4, CPz), parietal (P3, P4, Pz) and occipital cortex (O1, O2, O3). Repeated measures ANOVAs were conducted on the amplitude (baseline to peak) and peak latency (stimulus onset to peak), with group (S-REs, US-REs, UREs) as a between factor, and picture (high-energy food picture, low-energy food picture, neutral picture) and cortex as within factors. All the analysis was conducted by SPSS 17.0. *P*-value was computed for deviation in all analysis based on the Greenhouse-Geisser method. In addition, the Bonferroni method was used for the post-hoc comparisons.

## Results

### Behavioral data

Results showed there was no significant difference in levels of hungry and fullness pre- (F_(2,39)_ =. 889, *p* =. 419; F_(2,39)_ =. 640, *p* =. 533) and post-experiment (F_(2,39)_ =. 378, *p* =. 688; F_(2,39)_ = 3.197, *p* =. 052) in S-REs, US-REs and UREs (See Table 2). Trials with incorrect responses, too fast (RTs than 200 ms) or too slow (RTs more than 1000ms) were excluded. On the mean reaction times (RT), 3 (picture: High-energy food pictures, low-energy food pictures, neutral pictures) × 3(group: S-REs, US-REs, UREs) repeated measures ANOVA showed main effect of picture (F_(1.806, 70.439)_ = 18.437, *p* =. 001). Extra main or interaction effect was not significant. Post-hoc test showed that the RTs of high-energy food picture was longer compared to the low-energy food picture and neutral picture (*t*_(38)_ = 5.577, *p* =. 001; *t*_(38)_ = 4.524, *p* =. 001). Furthermore, no significant effects on the accuracy were found.

**Table 2:** The descriptive and AVOVA results on the hungry/fullness level pre- and post-experiment.

Hungry& fullness	Group	M ± SD	F value & *p* value
H_Pr	S-RE	38.93±5.35	
	US-RE	49.14±6.60	F_(2,39)_ =. 889, *p* =. 419
	URE	39.29±6.42	
F_Pr	S-RE	45.79±6.88	
	US-RE	46.43±5.80	F_(2,39)_ =. 640, *p* =. 533
	URE	54.93±6.48	
H_Po	S-RE	57.43±2.26	
	US-RE	63.93±5.80	F_(2,39)_ =. 378, *p* =. 688
	URE	63.57±5.51	
F_Po	S-RE	56.43±6.84	
	US-RE	37.50±5.26	F_(2,39)_ = 3.197, *p* =. 052
	URE	37.50±6.13	

Note: H_Pr = the hungry state pre-experiment; F_Pr = the fullness state pre-experiment; H_Po = the hungry state post experiment; F_Po = the fullness state post experiment.

### ERP Data

**Fig 1:**
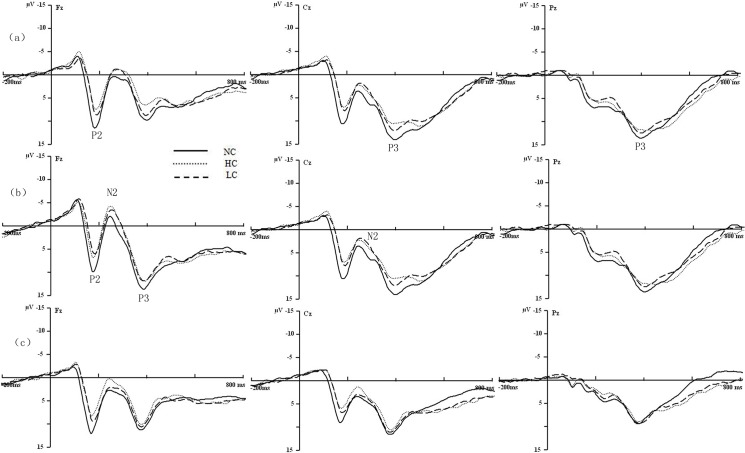
Grand average waveform of the stimulus-locked ERPs for P2, N2 and P3 at Fz, Cz and Pz in S-RE (a), US-RE (b) and URE(c).

Results of repeated measures ANOVA of 3 (picture: High-energy food pictures, low-energy food pictures, neutral pictures)×3(group: S-REs, US-REs, UREs) ×4 (cortex: Frontal F3, F4, Fz; frontal-central FC3, FC4, FCz; central C3, C4, Cz; central-parietal CP3, CP4, CPz) on P2 amplitude showed main effects of picture (F_(1.089, 42.486)_ = 7.531, *p* =. 007) and cortex (F_(1.580, 61.616)_ = 7.220, *p* =. 003). The post-hoc test showed high-energy and low energy food pictures elicited smaller P2 amplitude compared to the neutral pictures (*t*_(38)_ = 4.358, *p* =. 001; *t*_(38)_ = 3.203, *p* =. 035), and high-energy food pictures were not significantly different from low-energy food pictures on the P2 amplitude. Post-hoc test on the cortex showed that P2 amplitude in the frontal cortex were larger than the central cortex (*t*_(37)_ = 2.853, *p* =. 041), P2 amplitude in the frontal-central cortex was larger than the central and parietal cortex (*t*_(37)_ = 4.132, *p* =. 001; *t*_(37)_ = 3, *p* =. 028). Moreover, interaction of picture and cortex was also significant (F_(2.363,92.145)_ = 61.452, *p* =. 001)(Fig 1 and Table 3). Simple effect analysis showed for the high-energy food pictures, P2 amplitude in the frontal-central cortex was larger than in frontal, central and central-parietal cortex (F_(3,37)_ = 11.284, *p* =. 001; F_(3,37)_ = 8.120, *p* =. 001; F_(3,37)_ = 11.942, *p* =. 001). For the low-energy food pictures, P2 amplitude in the frontal-central cortex was larger than in frontal-central, central and central-parietal cortex (F_(3,37)_ = 8.777, *p* =. 001; F_(3,37)_ = 11.497, *p* =. 001; F_(3,37)_ = 2.863, *p* =. 007). For the neutral pictures, P2 was larger in the central cortex than the frontal, frontal-central and central-parietal cortex (F_(3,37)_ = 4.824, *p* =. 001; F_(3,37)_ = 9.412, *p* =. 001; F_(3,37)_ = 8.192, *p* =. 001).

**Table 3:** The differences of P2, N2 and P3 components in S-RE, U-RE and UE at Fz, Cz and Pz electrodes.

			P2(M±SD)		N2(M±SD)		P3(M±SD)	
Cortex	Picture	Group	Latency	Amplitude	Latency	Amplitude	Latency	Amplitude
Fz	HC	S-RE	173.60±12.61	7.25±4.97	254.67±20.60	-3.32±5.10	427.38±62.82	9.47±5.96
		US-RE	165.64±6.74	7.42±6.39	244.55±23.60	-6.61±6.81	401.69±66.21	14.21±8.44
		URE	170.92±15.97	9.21±5.99	252.00±23.44	-2.24±7.13	373.54±23.68	11.42±6.22
	LC	S-RE	176.27±9.35	11.59±5.62	251.60±25.75	-1.88±4.72	400.15±48.56	11.26±4.15
		US-RE	170.55±8.63	11.08±6.51	236.55±21.45	-3.10±7.62	388.15±56.29	15.42±9.65
		URE	176.77±15.50	12.91±6.33	251.85±25.53	-.75±6.83	373.38±40.32	12.95±7.21
	NC	S-RE	176.67±17.88	8.24±5.27	253.73±17.42	-2.96±4.69	409.85±56.22	11.68±6.47
		US-RE	169.64±15.33	6.80±5.22	247.82±25.91	-4.91±6.73	368.92±29.83	10.59±5.35
		URE	172.92±19.25	10.38±5.59	256.62±22.17	-.98±6.39	367.23±19.55	13.45±7.89
	HC	S-RE	172.27±15.94	7.42±5.17	242.53±22.88	.82±4.83	436.00±55.13	11.98±6.70
		US-RE	172.18±22.35	7.89±5.09	238.55±24.46	-1.00±4.33	398.00±66.50	13.90±6.76
		URE	169.54±17.51	8.52±4.79	237.08±23.19	-.09±6.13	378.15±38.89	19.07±4.38
Cz	LC	S-RE	170.13±15.46	11.53±5.30	233.73±20.04	2.45±4.59	419.54±47.87	15.03±6.51
		US-RE	163.82±17.54	11.68±4.87	235.27±26.48	1.95±4.64	378.15±46.99	16.23±4.48
		URE	169.85±18.81	11.68±5.00	239.38±26.31	1.31±5.56	365.54±19.94	20.48±7.46
	NC	S-RE	171.60±14.25	7.86±5.26	243.33±18.09	.75±4.34	411.23±47.86	16.67±6.36
		US-RE	164.55±12.84	7.67±4.14	241.45±28.14	-.04±3.68	388.46±58.58	14.29±5.34
		URE	166.15±13.99	9.30±4.25	254.92±28.43	.80±5.24	387.85±52.88	18.22±6.41
	HC	S-RE	-	-	-	-	416.46±63.59	15.08±5.81
		US-RE	-	-	-	-	410.00±66.49	14.08±1.95
		URE	-	-	-	-	378.15±42.16	20.23±4.64
Pz	LC	S-RE	-	-	-	-	413.08±49.86	14.94±5.60
		US-RE	-	-	-	-	366.15±36.42	14.88±2.35
		URE	-	-	-	-	363.85±18.27	21.25±5.20
	NC	S-RE	-	-	-	-	388.00±32.37	15.62±5.71
		US-RE	-	-	-	-	383.54±38.94	14.16±2.65
		URE	-	-	-	-	366.46±27.76	19.50±4.69

Note: HC = high-energy food picture, LC = low-energy food picture, NC = Neutral picture.

Similar repeated measures ANOVA was conducted on the P2 latency, and results showed main effects of picture (F_(1.259, 49.099)_ = 38.674, *p* =. 001) and cortex (F_(1.937, 75.546)_ = 7.546, *p* =. 001). The interaction effects of picture and cortex (F_(3.518, 49.099)_ = 2.528, *p* =. 047), picture and group(F_(2.917,113.746)_ = 18.049, *p* =. 001), were also significant. Post-hoc test showed P2 latency in frontal cortex was shorter than in frontal-central, central-parietal cortex (*t*_(37)_ = 3.142, *p* =. 020; *t*_(37)_ = 3.065, *p* =. 024; *t*_(37)_ = 4.381, *p* =. 001), and P2 latencies of high-energy food and low-energy food pictures were shorter compared to the neutral pictures (*t*_(38)_ = 6.284, *p* =. 001; *t*_(38)_ = 7.034, *p* =. 001). Simple effect analysis on the interaction of picture and group showed P2 latency of high-energy food pictures was shorter compared to the neutral pictures (F_(2,38)_ = 4.391, *p* =. 001) in S-REs. P2 latencies of high-energy food and low-energy food pictures were shorter in contrast to the neutral pictures(F_(2,38)_ = 4.765, *p* =. 001; F_(2,38)_ = 4.744, *p* =. 001), and P2 latency of the high-energy food picture was shorter than low-energy food picture in US-REs (F_(2,38)_ = 2.492, *p* =. 017). P2 latency of the high-energy food was shorter than the neutral picture in UREs (F_(2,38)_ = 2.256, *p* =. 030). Simple effect analysis on the picture and cortex showed that for the high-energy food pictures, P2 latency was shorter in frontal-central cortex compared to frontal, central and central-parietal cortex (F_(3,37)_ = 2.897, *p* =. 006; F_(3,37)_ = 8.499, *p* =. 006; F_(3,37)_ = 3.466, *p* =. 001). For the low-energy food pictures, P2 latency was shorter in frontal cortex compared to frontal-central and parietal cortex (F_(3,37)_ = 7.336, *p* =. 001; F_(3,37)_ = 3.900, *p* =. 001). For the neutral pictures, P2 was shorter in the central cortex compared to the frontal, frontal-central and central-parietal cortex (F_(3,37)_ = 2.181, *p* =. 035; F_(3,37)_ = 2.940, *p* =. 005; F_(3,37)_ = 5.408, *p* =. 001).

For N2 amplitude, 3 (picture: High-energy food pictures, low-energy food pictures, neutral pictures)×3(group: S-REs, US-REs, UREs)×4(cortex: Frontal F3, F4, Fz; frontal-central FC3, FC4, FCz; central C3, C4, Cz; central-parietal CP3,CP4,CPz) repeated measures ANOVA showed main effects of picture and cortex (F_(1.226,47.806)_ = 12.603, *p* =. 001; F_(1.226,47.806)_ = 12.603, *p* =. 001), and interaction of picture and cortex (F_(1.226,47.806)_ = 12.603, *p* =. 001) (Fig 1 and Table 3). Post-hoc test showed the high- and low-energy food pictures elicited more negative N2 amplitude than the neutral pictures (*t*_(38)_ = 6.779, *p* =. 001; *t*_(38)_ = 5.197, *p* =. 001), the high-energy food picture elicited larger N2 amplitude compared to the low-energy food pictures (*t*_(38)_ = 7.940, *p* =. 001). N2 amplitude was smaller in the frontal than in the frontal-central, central and central-parietal cortex (*t*_(38)_ = 7.114, *p* =. 001; *t*_(38)_ = 7.069, *p* =. 001; *t*_(38)_ = 6.711, *p* =. 001). Simple effect analysis showed that for the high-energy food pictures, N2 amplitude in frontal cortex was larger than central-parietal cortex (F_(3,37)_ = 7.009, *p* =. 001). For the low-energy food pictures, N2 amplitude was larger in the central-parietal cortex than frontal cortex (F_(3,37)_ = 5.891, *p* =. 001), frontal-central cortex(F_(3,37)_ = 6.592, *p* = 0.001) and central cortex (F_(3,37)_ = 4.694, *p* = 0.001). For the neutral pictures, N2 amplitude was larger in central-parietal cortex than frontal cortex, frontal-central and parietal cortex (F_(3,37)_ = 4.694, *p* =. 001; F_(3,37)_ = 2.181, *p* =. 035; F_(3, 37)_ = 2.075, *p* =. 041). Results from N2 latency showed a main effect of picture (F_(1.226, 47.806)_ = 12.603, *p* =. 001), and interaction effect of picture and cortex (F_(3.169, 123.589)_ = 7.576, *p* =. 001). Post-hoc test showed that N2 latency of the high-energy food picture was longer than the low-energy food pictures and neutral pictures (*t*_(38)_ = 4.932, *p* =. 001;*t*_(38)_ = 3.736, *p* =. 002). Simple effect analysis showed that N2 latency of the high-energy food picture was shorter in frontal-central cortex than central cortex (F_(3,37)_ = 3.292, *p* =. 002). For the low-energy food pictures, N2 latency was shorter in central-parietal cortex compared to frontal-central cortex (F_(3,37)_ = 4.348, *p* =. 001). For neutral pictures, N2 latency was shorter in central cortex compared to frontal and central-parietal cortex (F_(3,37)_ = 2.850, *p* =. 006;F_(3,37)_ = 2.050, *p* =. 047).

On P3 amplitude, repeated measures of 3 (picture: High-energy food pictures, low-energy food pictures, neutral pictures)×3(group: S-REs, US-REs, UREs)×5 (cortex: Frontal F3, F4, Fz; frontal-central FC3, FC4, FCz; central C3, C4, Cz; central-parietal CP3, CP4, CPz; parietal P3, P4, Pz) showed main effects of picture and cortex (F_(1.118,43.596)_ = 10.240, *p* =. 002; F_(1.518,59.191)_ = 8.435, *p* =. 002), interaction of picture and group (F_(2.762,43.596)_ = 11.478, *p* =. 001). Post-hoc test showed P3 amplitude in the parietal cortex was larger than the frontal, frontal-central, central and parietal cortex (*t*_(36)_ = 1.710, *p* =. 001; *t*_(36)_ = 2.294, *p* =. 001; *t*_(36)_ = 43.421, *p* =. 001; *t*_(36)_ = 5.214, *p* =. 001), and the low-energy food pictures and neutral pictures elicited larger P3 amplitude than the high-energy food pictures (*t*_(38)_ = 3.735, *p* =. 002; *t*_(38)_ = 3.263, *p* =. 007). Simple effect analysis showed that in S-REs, the low-energy food pictures and neutral pictures elicited larger P3 amplitude than the high-energy food pictures (F_(2,38)_ = 2.398, *p* = 0.021; F_(2,38)_ = 2.701, *p* =. 010), the neutral pictures elicited larger P3 amplitude than the low-energy food pictures (F_(2,38)_ = 2.578, *p* =. 014). For US-REs, the neutral pictures elicited larger P3 amplitude than the high- and low-energy food pictures (F_(2,38)_ = 2.387, *p* =. 032; F_(2,38)_ = 2.163, *p* =. 047). For UREs, there was no significant difference in the picture (Fig 1 and Table 3).

Similar repeated measures ANOVA was conducted on the P3 latency, and results only showed interaction effect of picture and cortex (F_(3.403,132.724)_ = 4.595, *p* =. 003). Simple effect analysis showed that for the high-energy food pictures, P3 latency was longer in central-parietal cortex compared to the frontal-central, central and parietal cortex (F_(4,36)_ = 2.255, *p* =. 030; F_(4,36)_ = 2.263, *p* =. 029; F_(4,36)_ = 2.397, *p* =. 021). For the low-energy food pictures, P3 latency was longer in parietal cortex compared to central and central-parietal cortex (F_(4,36)_ = 2.351, *p* =. 024; F_(4,36)_ = 2.048, *p* =. 048). For the neutral pictures, P3 amplitude was longer in the central cortex than the frontal, frontal-central, central-parietal and parietal cortex (F_(4,36)_ = 2.322, *p* =. 026; F_(4,36)_ = 2.164, *p* =. 037; F_(4,36)_ = 3.317, *p* =. 002; F_(4,36)_ = 2.918, *p* =. 006).

## Discussion

### Behavioral response

The present study shows that S-REs and US-REs did not behaviorally differ in specific inhibition ability, which is not consistent with available findings that REs are deficient in terms of their specific inhibition ability [5, 11, 15]. This finding, however, was consistent with the findings of Hachl, Hempel, and Pietrowsky (2003)[16]. The probable interpretation is that S-REs and UREs intentionally allow for the regulation of the inhibition ability [27].

### Electrophysiological response

Results showed that the P2 latency of the high-energy food picture was shorter than that of the neutral pictures in S-REs. The P2 latencies of the high- and low-energy food pictures were shorter compared with that of the neutral pictures in US-REs, which tested our hypothesis. P2 indicated the perception analysis of the stimuli attributes in the brain [28]. Thus, S-REs rapidly completed the perception analysis of high-energy food pictures, while US-REs easily finished the perception analysis of both high- and low-energy food pictures. As such, S-REs were found to be more sensitive to high-energy food and US-REs were more sensitive to food information regardless of energy due to their long dieting activity [29, 30]. Moreover, the P2 amplitudes of the high- and low-energy food pictures were smaller than the neutral pictures, which were related to the survival value of the food cues [31].

Moreover, results reflected that S-REs and US-REs show no difference in the N2 component, although the difference of N2 amplitude in S-REs, US-REs, and UREs reached a numerical edge. This finding does not support our hypothesis, but is consistent with Watson and Garvey’s (2013) [19] study, wherein food cues elicited larger N2 component than did neutral cues in both REs and UREs. N2 reflected the conflict detection [32], and the closer to the pre-frontal cortex, the stronger the conflict monitor [33], indicating that S-REs did not differ from US-REs in conflict monitor the target stimuli. This result was not consistent with previous findings [3,34], which was probably caused by the fact that S-REs and US-REs scored high in the dimension of restraint dietary and presumably performed normally in daily eating behaviors except for some specific conditions [10,15]. Another probable reason is that both S-REs and US-REs are chronic dieters [30] and have developed an enhanced sensitivity to food cues because of the long-term diet activity.

Furthermore, the P3 amplitudes of the low-energy and neutral pictures were found to be larger than the high-energy food pictures, the P3 amplitudes of the neutral pictures were larger than the low-energy food pictures in S-REs, and P3 amplitude of the neutral pictures was larger than those of the high-energy and low-energy food pictures in US-REs, which is not consistent with previous research [18, 19].

Given that the tasks were different from that in Watson and Garvey’s (2013) study and similar to that in Babiloni et al. (2011), the current study’s results are analyzed along with that of Babiloni et al. (2011). Babiloni et al. (2011) showed similar P3 amplitude evoked by the food pictures between normal-weight successful dieters and non-dieters, but larger frontal-parietal P3 component among successful dieters [18]. Although there were some discrepancies, the visual oddball paradigm was also used in the current study. First, in Balboni et al.’s (2011), participants were asked to respond only to the deviant stimuli, but in the current study, participants were required to react to the standard and deviant stimuli. Second, the standard and deviant stimuli were the same in their study, with the deviant stimuli being the “fattening” standard stimuli. However, in the present study, we used different pictures for the standard and deviant stimuli, and the deviant stimuli included high-energy food pictures, low-energy food pictures, and neutral pictures. Finally, the current study utilized three groups (S-REs, US-REs, UREs), whereas the previous study used successful dieter and non-dieters.

In processing food cues, the P3 component was associated with response inhibition [16]. Therefore, only US-REs were deficit in the inhibit reaction to the nonfood and food cues (high- and low-energy food) and S-RES were only deficit in the response inhibition to the high-energy food cues. Previous studies demonstrated that S-REs and US-REs used different processing strategies in the food exposure condition [35]. S-REs usually employed the flexible control strategy, depending on the specific food. However, US-REs often employed strict control strategies to treat all types of food. The flexible strategy benefited the successful dieting in the long-term, and the strict control strategy brought short-term effects and finally failed to overeat [20, 35]. Furthermore, REs were over-confident about successfully inhibiting their response when facing palatable food, but often failed in the end [36].

The current study has several limitations. First, the inhibition deficit was analyzed based on the temporal course of food cues processing. Future studies should thus use a combination of the technologies of ERP and functional magnetic resonance imaging to explore the inhibition ability among REs. Second, participants were grouped based on DEBQ and TFEQ measures. However, the actual eating behavior probably differed from the performances indicated by the items. Further studies should take into consideration the actual eating behaviors when grouping subjects. Finally, different people have different appetite or eating habits [37]. Thus, future studies should use the individualized food pictures to control this effect.

## Conclusion

This study was among the first to examine the neural correlates of inhibition ability in S-REs, US-REs, and unrestrained eaters. The results showed that S-REs were deficient in terms of inhibiting high-energy food pictures and US-REs were deficient in terms of inhibiting both high- and low-energy food pictures. The results suggest that S-REs and US-REs had different inhibition abilities, which is an important factor in successful dieting.

## Supporting Information

S1 Dataset (XLS)
