# Correction: Inhibition Ability of Food Cues between Successful and Unsuccessful Restrained Eaters: A Two-Choice Oddball Task

**DOI:** 10.1371/journal.pone.0133942

**Published:** 2015-07-20

**Authors:** Fanchang Kong, Yan Zhang, Hong Chen

There is information missing from funding section of this paper. The funding section should read as follows: This research was supported and granted by Chinese National Natural Science Foundation (31170981 & 31200787), China Postdoctoral Science Foundation funded project (2013M531712), The Ministry of Education-China Mobile Foundation (MCM20130601), and Fundamental Research Funds for the Central Universities, China (CCNU15A05048), Natural Science Foundation of Hubei Province of China (2015CFB534), Humanities and Social Science Project of Educational Commission of Hubei Province of China(15Z033). The funders had no role in study design, data collection and analysis, decision to publish, or preparation of the manuscript.
